# Prognostic value of serum total bilirubin-lipid ratios in patients with IgA nephropathy

**DOI:** 10.3389/fendo.2026.1898387

**Published:** 2026-09-16

**Authors:** Siwen Wang, Yilin Chen, Huan Zhou, Lingqiu Dong, Yi Tang, Jiaxing Tan, Wei Qin

**Affiliations:** Division of Nephrology, Department of Medicine, West China Hospital, Sichuan University, Chengdu, Sichuan, China

**Keywords:** bilirubin-lipid ratio, composite renal outcome, end-stage renal disease, IgA nephropathy, prognosis

## Abstract

**Background:**

Reliable prognostic biomarkers are needed for IgA nephropathy (IgAN), a common glomerular disease frequently progressing to end-stage renal disease (ESRD). Given bilirubin’s antioxidant and dyslipidemia’s pro-inflammatory roles, we hypothesized that TBIL-lipid ratios would serve as novel composite biomarkers associated with renal outcomes in IgAN.

**Methods:**

We evaluated four serum bilirubin-lipid ratios (TBIL/Triglyceride, TBIL/Cholesterol, TBIL/HDL, TBIL/LDL) using Cox regression, restricted cubic spline (RCS), and linear mixed-effects (LME) models in biopsy-proven IgAN patients.

**Results:**

Of the 1352 patients included in this study, 283 (20.93%) developed composite renal outcome (≥30% decline in estimated glomerular filtration rate [eGFR] or ESRD). Multivariable Cox analysis showed that all four ratios were independently associated with reduced outcome risk: TBIL/Triglyceride (HR = 0.972, P = 0.025), TBIL/Cholesterol (HR = 0.849, P = 0.003), TBIL/HDL (HR = 0.970, P = 0.030), and TBIL/LDL (HR = 0.926, P = 0.004). Although the proportional hazards assumption was violated (all P < 0.05), time-dependent and piecewise Cox models confirmed that the protective effects remained significant in the early phase (≤3.13 years) but attenuated thereafter. RCS analyses demonstrated linear inverse associations for TBIL/Triglyceride, TBIL/Cholesterol, and TBIL/HDL, whereas TBIL/LDL exhibited a threshold-saturation pattern. These associations were more pronounced in those with advanced tubulointerstitial lesions (Oxford T1/T2). In LME models, all four ratios were positively associated with longitudinal eGFR levels (TBIL/Triglyceride: Estimate = 0.318, P = 0.002; TBIL/Cholesterol: Estimate = 1.242, P = 0.004; TBIL/HDL: Estimate = 0.289, P = 0.007; TBIL/LDL: Estimate = 0.446, P = 0.031), with associations remaining stable during follow-up.

**Conclusion:**

Higher serum bilirubin-lipid ratios, particularly TBIL/Cholesterol, were independently associated with lower risk of adverse renal outcomes and better preserved renal function in IgAN patients. These readily available biomarkers may provide additional value for risk stratification in clinical practice.

## 1 Introduction

IgA nephropathy (IgAN) is the most common primary glomerular disease worldwide, characterized by the deposition of IgA immune complexes in the glomerular mesangium (1, 2). The pathogenesis of IgAN involves complex interactions among immune dysregulation, chronic inflammation, and oxidative stress, which collectively contribute to glomerular injury and disease progression (2, 3). As a leading cause of end-stage renal disease (ESRD), IgAN imposes a substantial burden on global healthcare systems, necessitating the identification of reliable prognostic biomarkers to stratify patient risk and guide clinical management (4, 5).

Serum bilirubin and lipid profiles have gained attention as potential prognostic indicators for kidney diseases (6–8). Historically regarded as a metabolic waste product from heme catabolism, serum total bilirubin (TBIL) is increasingly recognized as a potent endogenous antioxidant with anti-inflammatory and cytoprotective properties (9). Mild elevation of TBIL levels has been associated with preserved renal function and reduced cardiovascular risk in various kidney diseases (7, 10, 11). The protective mechanisms of bilirubin are primarily attributed to its ability to scavenge reactive oxygen species, inhibit lipid peroxidation, and modulate inflammatory pathways (9, 12).Conversely, dyslipidemia—characterized by abnormalities in triglycerides, total cholesterol, high-density lipoprotein (HDL), and low-density lipoprotein (LDL)—is highly prevalent in chronic kidney disease (CKD) (13). Lipid abnormalities play a pivotal role in promoting systemic inflammation, oxidative stress, and progressive glomerulosclerosis via direct lipid toxicity (14).

To better capture the interplay between antioxidant defense and lipid-induced inflammation or fibrotic stress, we propose the use of serum TBIL-lipid ratios as novel composite biomarkers. These ratios (namely TBIL/Triglycerides, TBIL/Cholesterol, TBIL/HDL, and TBIL/LDL) integrate TBIL and individual lipid fractions, potentially offering a more comprehensive reflection of the underlying pathophysiological mechanisms compared to single biomarkers. Despite their theoretical benefits, the prognostic utility of TBIL-lipid ratios in IgAN remains unexplored. Building upon this knowledge gap, the present study aimed to evaluate the longitudinal associations between baseline TBIL-lipid ratios and both long-term renal outcomes and dynamic estimated glomerular filtration rate (eGFR) trajectories in a well-characterized IgAN cohort. We hypothesized that these novel TBIL-lipid ratios may serve as robust and independent predictors for IgAN progression.

## 2 Method

### 2.1 Study population and design

This single-center, retrospective cohort study was conducted at West China Hospital, Sichuan University. Adult patients (age ≥ 18 years) newly diagnosed with IgA nephropathy via renal biopsy between 2009 and 2025 were consecutively enrolled. The diagnosis of IgAN was confirmed by standard pathological assessment based on immunofluorescence demonstrating predominant mesangial IgA deposition. Rigorous exclusion criteria were applied to ensure the inclusion of patients with primary IgAN and to minimize confounding from comorbid conditions. Patients were excluded if they met any of the following criteria: 1) secondary IgAN caused by disorders such as Henoch-Schönlein purpura, systemic lupus erythematosus, liver cirrhosis, rheumatoid arthritis, or ankylosing spondylitis; 2) a history of other primary kidney diseases (e.g. diabetic nephropathy, membranous nephropathy); 3) prior kidney transplantation or receipt of renal replacement therapy; 4) active, chronic, or latent infections such as viral hepatitis, tuberculosis, or human immunodeficiency virus (HIV); 5) any active or historical malignancy; 6) significant hepatic dysfunction, defined as ALT or AST > 3× upper limit of normal (ULN) with total bilirubin > 2× ULN, based on Hy’s Law criteria (15); and 7) missing data for any baseline or follow-up variables required for all analyses. The study protocol was reviewed and approved by the Ethics Committee of West China Hospital, Sichuan University (Approval No. 2019-33). A total of 1821 patients with biopsy-proven IgA nephropathy were initially assessed for eligibility. After exclusion of 469 patients according to exclusion criteria, 1352 patients were finally included in the present study (**Supplementary Figure S1**).

### 2.2 Data collection

Baseline clinical, demographic, and laboratory data were systematically extracted from the hospital’s electronic medical record system at the time of renal biopsy. The following variables were collected:

Demographics and Clinical History: Gender, age, history of diabetes mellitus (defined as fasting blood glucose ≥ 7.0 mmol/L, or random blood glucose ≥11.1 mmol/L) (16), hypertension (defined as blood pressure ≥140/90 mmHg or current use of antihypertensive drugs) (17), smoking status, alcohol consumption, and initial treatment regimen (classified as best supportive care, corticosteroids, immunosuppressants, or targeted drugs).

Pathological Parameters: Oxford MEST-C classification scores, including mesangial hypercellularity (M), endocapillary hypercellularity (E), segmental glomerulosclerosis (S), and tubular atrophy/interstitial fibrosis (T). The crescentic score (C) was also recorded.

Laboratory Parameters: Fasting venous blood samples were analyzed for: serum total bilirubin (TBIL, μmol/L), alanine aminotransferase (ALT, U/L), aspartate aminotransferase (AST, U/L), estimated glomerular filtration rate (eGFR, mL/min/1.73 m², calculated using the Chronic Kidney Disease Epidemiology Collaboration equation), serum uric acid (UA, μmol/L), triglycerides (mmol/L), total cholesterol (mmol/L), high-density lipoprotein cholesterol (HDL, mmol/L), low-density lipoprotein cholesterol (LDL, mmol/L), and 24-hour urine protein excretion (g/24h). Serum total bilirubin was measured using the Mindray Total Bilirubin (T-Bil) Assay Kit (vanadate oxidation method). Triglycerides, total cholesterol, HDL, and LDL were measured using Roche enzymatic colorimetric assays, with HDL and LDL determined by direct homogeneous assays rather than by calculation.

### 2.3 Renal endpoint

The primary endpoint of the study was a composite renal outcome, defined as a ≥ 30% decline in eGFR from baseline or progression to ESRD, which was characterized by one of the following criteria: (1) sustained eGFR < 15 mL/min/1.73 m², (2) initiation of maintenance renal replacement therapy (dialysis), or (3) kidney transplantation. The secondary endpoint was the longitudinal change in eGFR over time.

### 2.4 Statistical analysis

A complete case analysis approach was adopted. Patients with missing values for any of the core exposure or covariate variables were excluded from the analysis based on prespecified exclusion criterion 7). No imputation methods were applied. The normality of continuous variables was assessed using the Shapiro-Wilk test. Normally distributed variables are reported as mean ± standard deviation, non-normally distributed variables as median (interquartile range, IQR), and categorical variables as frequency (percentage). The four primary exposure variables were calculated as TBIL-lipid ratios: TBIL/Triglyceride, TBIL/Cholesterol, TBIL/HDL, and TBIL/LDL. For Kaplan-Meier survival analyses, each ratio was dichotomized into low and high groups based on the cohort’s median values. Univariable and multivariable Cox proportional hazards models were constructed to determine hazard ratio (HR) and 95% confidence interval (CI). Before conducting Cox regression analyses, we screened candidate covariates for inclusion using the change-in-estimate (CIE) analysis, wherein variables were classified as confounders if they altered the HR of bilirubin-lipid ratios by ≥ 10% (18, 19). To evaluate the incremental predictive value of the ratios, Harrell’s C-index, continuous net reclassification improvement (NRI), and integrated discrimination improvement (IDI) were calculated. Schoenfeld residual tests were used to assess the proportional hazards (PH) assumption for each ratio. Time-dependent Cox regression with Time × Ratio interaction terms and piecewise Cox models stratified at the median follow-up time were used as sensitivity analyses. Knot selection for restricted cubic splines (RCS) was guided by the Akaike information criterion (AIC), with models of 3 to 5 knots evaluated. When ΔAIC between models was less than 2, the more parsimonious model with fewer knots was selected to avoid overfitting (20). Receiver operating characteristic (ROC) curves were generated and optimal cut-off values were determined using the Youden index. Linear mixed-effects (LME) models (nlme package) were used to examine the longitudinal associations between TBIL-lipid ratios and repeated measurements of eGFR over time. Statistical significance was defined as a two-sided p-value < 0.05. All analyses were performed in R software (version 4.3.3).

## 3 Results

### 3.1 Baseline characteristics of the study population

A total of 1352 biopsy-proven IgA nephropathy patients were included in the final analysis. During a median follow-up of 3.13 years (IQR: 1.59–5.70; maximum 13.43 years), 283 patients (20.93%) reached the composite renal outcome, defined as a ≥30% decline in eGFR or progression to ESRD. Baseline characteristics stratified by outcome status are presented in **Table 1**. The median age of the cohort was 34 years (IQR: 28–43), with a higher proportion of female patients (58.21%). Compared with patients who remained event-free, those who reached the composite endpoint were older (median 36 vs. 33 years, P = 0.015), more likely to have hypertension (44.52% vs. 31.90%, P < 0.001), and more frequently reported a history of smoking (21.55% vs. 12.44%, P < 0.001). No significant differences were observed in the prevalence of diabetes or alcohol consumption between the two groups. Treatment regimens differed significantly (P = 0.009), with a higher proportion of patients in the composite outcome group receiving immunosuppressants (27.21% vs. 19.08%).

**Table 1:** Baseline characteristics of the study population.

Variables	Overall	No composite renal outcome	Composite renal outcome	P-value
(median [IQR])	N=1352	N=1069	N=283	
Gender (%)				0.327
Female	787 (58.21)	630 (58.93)	157 (55.48)	
Male	565 (41.79)	439 (41.07)	126 (44.52)	
Age (years)	34.00 [28.00, 43.00]	33.00 [28.00, 43.00]	36.00 [29.00, 44.00]	0.015
Diabetes (%)	41 (3.03)	28 (2.62)	13 (4.59)	0.127
Hypertension (%)	467 (34.54)	341 (31.90)	126 (44.52)	<0.001
Smoking (%)	194 (14.35)	133 (12.44)	61 (21.55)	<0.001
Alcohol (%)	225 (16.64)	171 (16.00)	54 (19.08)	0.25
Treatment (%)				0.009
Best Supportive Care	560 (41.42)	457 (42.75)	103 (36.40)	
Glucocorticoids	503 (37.20)	400 (37.42)	103 (36.40)	
Immunosuppressant	281 (20.78)	204 (19.08)	77 (27.21)	
Targeted Drugs	8 (0.59)	8 (0.75)	0 (0.00)	
Lipid-lowering (%)	194 (14.35)	150 (14.03)	44 (15.55)	0.581
RAAS (%)	635 (46.97)	520 (48.64)	115 (40.64)	0.020
SGLT2 (%)	78 (5.77)	63 (5.89)	15 (5.30)	0.813
Oxford Classification (%)				
M0	305 (22.56)	249 (23.29)	56 (19.79)	0.240
M1	1047 (77.44)	820 (76.71)	227 (80.21)	
E0	1285 (95.04)	1014 (94.86)	271 (95.76)	0.639
E1	67 (4.96)	55 (5.14)	12 (4.24)	
S0	408 (30.18)	331 (30.96)	77 (27.21)	0.250
S1	944 (69.82)	738 (69.04)	206 (72.79)	
T0	1088 (80.47)	902 (84.38)	186 (65.72)	<0.001
T1	222 (16.42)	147 (13.75)	75 (26.50)	
T2	42 (3.11)	20 (1.87)	22 (7.77)	
C0	1114 (82.40)	885 (82.79)	229 (80.92)	0.367
C1	213 (15.75)	167 (15.62)	46 (16.25)	
C2	25 (1.85)	17 (1.59)	8 (2.83)	
TBIL (umol/L)	9.50 [7.20, 13.00]	9.70 [7.30, 13.20]	9.10 [6.95, 12.10]	0.088
ALT (U/L)	17.00 [12.00, 26.00]	17.00 [12.00, 26.00]	18.00 [14.00, 26.00]	0.012
AST (U/L)	19.00 [16.00, 24.00]	19.00 [16.00, 24.00]	20.00 [17.00, 25.00]	0.049
eGFR (ml/(min·1.73m²))	87.47 [64.37, 109.85]	90.54 [67.74, 112.13]	75.32 [52.28, 101.80]	<0.001
UA (umol/L)	365.00 [303.00, 435.00]	358.00 [296.00, 426.70]	396.00 [333.90, 463.00]	<0.001
Triglyceride (mmol/L)	1.52 [1.08, 2.24]	1.49 [1.06, 2.21]	1.63 [1.13, 2.28]	0.047
Cholesterol (mmol/L])	4.82 [4.13, 5.62]	4.80 [4.12, 5.55]	4.89 [4.26, 5.92]	0.035
HDL (mmol/L)	1.34 [1.08, 1.65]	1.34 [1.07, 1.65]	1.37 [1.11, 1.65]	0.277
LDL (mmol/L)	2.82 [2.27, 3.44]	2.80 [2.26, 3.41]	2.92 [2.30, 3.67]	0.079
Proteinuria (g/24h)	1.29 [0.70, 2.43]	1.21 [0.69, 2.28]	1.80 [0.96, 3.00]	<0.001
TBIL/Triglyceride	6.37 [3.77, 10.52]	6.57 [3.80, 10.94]	5.76 [3.47, 9.37]	0.015
TBIL/Cholesterol	2.02 [1.41, 2.84]	2.03 [1.45, 2.87]	1.92 [1.26, 2.71]	0.018
TBIL/HDL	7.17 [4.92, 10.21]	7.23 [4.97, 10.30]	6.83 [4.61, 9.91]	0.059
TBIL/LDL	3.48 [2.34, 5.10]	3.52 [2.40, 5.18]	3.31 [2.14, 4.80]	0.033
Follow-up time (year)	3.13 [1.59, 5.70]	2.95 [1.42, 5.49]	4.18 [2.59, 6.40]	<0.001

IQR, interquartile range; ALT, alanine aminotransferase; AST, aspartate aminotransferase; RAAS, renin-angiotensin-aldosterone system; SGLT2, sodium-glucose cotransporter 2; TBIL, total bilirubin; eGFR, estimated glomerular filtration rate; UA, uric acid; HDL, high-density lipoprotein cholesterol; LDL, low-density lipoprotein cholesterol.

Regarding pathological features, patients who reached the composite outcome exhibited more severe tubular atrophy and interstitial fibrosis, as indicated by higher proportions of T1 (26.50% vs. 13.75%) and T2 (7.77% vs. 1.87%) lesions (P < 0.001). No significant differences were observed for M, E, S, or C scores between the two groups. At baseline, patients who subsequently developed the composite outcome had significantly lower eGFR (75.32 vs. 90.54 mL/min/1.73 m², P < 0.001), higher serum uric acid levels (396.00 vs. 358.00 μmol/L, P < 0.001), and greater proteinuria (1.80 vs. 1.21 g/24h, P < 0.001). Serum total bilirubin levels were marginally lower in the composite outcome group (9.10 vs. 9.70 μmol/L, P = 0.088). Regarding lipid profiles, patients who reached the composite outcome had significantly higher triglycerides (1.63 vs. 1.49 mmol/L, P = 0.047) and total cholesterol (4.89 vs. 4.80 mmol/L, P = 0.035), whereas HDL and LDL levels did not differ significantly between groups.

Among the four bilirubin-lipid ratios, TBIL/Triglyceride (P = 0.015), TBIL/Cholesterol (P = 0.018), and TBIL/LDL (P = 0.033) were significantly lower in patients who progressed to the composite outcome, while TBIL/HDL showed a borderline significant difference (P = 0.059).

### 3.2 Prognostic value of bilirubin-lipid ratios for the composite renal outcome

Kaplan–Meier survival curves, stratified by median of each bilirubin–lipid ratio, are shown in **Supplementary Figure S2**. Patients in the low group of TBIL/Triglyceride, TBIL/Cholesterol, and TBIL/LDL exhibited a significantly higher cumulative risk of the composite renal outcome compared with those in the high group (log-rank P = 0.002, 0.005, and 0.031, respectively). In contrast, no significant difference in event-free survival was observed between the high and low groups of TBIL/HDL (log-rank P = 0.722).

Confounder assessment using the CIE analysis showed that none of the candidate covariates altered the HR for the bilirubin-lipid ratios by at least 10% (**Supplementary Figure S3**), suggesting that the observed associations were not materially influenced by any single covariate. Nevertheless, to account for clinically relevant factors and covariates previously associated with renal prognosis in IgAN, multivariable Cox models were adjusted for age, gender, eGFR, serum uric acid, proteinuria, diabetes, hypertension, and Oxford MEST-C pathological scores (Models 2–4). In the unadjusted model (Model 1), all four bilirubin-lipid ratios were significantly associated with a lower risk of the composite renal outcome (**Table 2**). These associations remained statistically significant after sequential adjustment (Models 2–4). In the fully adjusted model (Model 4), higher baseline levels of TBIL/Triglyceride (HR = 0.972, 95% CI: 0.947–0.996, P = 0.025), TBIL/Cholesterol (HR = 0.849, 95% CI: 0.763–0.946, P = 0.003), TBIL/HDL (HR = 0.970, 95% CI: 0.943–0.997, P = 0.030), and TBIL/LDL (HR = 0.926, 95% CI: 0.878–0.976, P = 0.004) were each independently associated with a lower risk of the composite renal outcome. In sensitivity analyses, we sequentially excluded patients with follow-up duration < 1 year, those with baseline eGFR < 30 mL/min/1.73 m², those with baseline proteinuria < 0.5 g/24h, and those receiving baseline lipid-lowering therapy. Additionally, to account for potential confounding by concomitant medications and hepatic function, we further adjusted for RAAS inhibitors, SGLT2 inhibitors, and liver enzymes (ALT and AST). The protective associations of the four bilirubin-lipid ratios remained consistent with the primary analysis (**Supplementary Table S1**).

**Table 2:** Association between bilirubin-lipid ratios and risk of composite renal outcome: univariable and multivariable Cox regression analyses.

	HR	95%CI	P value
TBIL/Triglyceride			
Model1	0.954	0.932-0.977	<0.001
Model2	0.959	0.936-0.982	0.001
Model3	0.974	0.950-0.998	0.034
Model4	0.972	0.947-0.996	0.025
TBIL/Cholesterol			
Model1	0.828	0.747-0.918	<0.001
Model2	0.823	0.741-0.914	<0.001
Model3	0.850	0.763-0.946	0.003
Model4	0.849	0.763-0.946	0.003
TBIL/HDL			
Model1	0.972	0.947-0.997	0.030
Model2	0.963	0.937-0.990	0.008
Model3	0.968	0.941-0.996	0.024
Model4	0.970	0.943-0.997	0.030
TBIL/LDL			
Model1	0.911	0.865-0.959	<0.001
Model2	0.912	0.866-0.960	<0.001
Model3	0.925	0.878-0.975	0.004
Model4	0.926	0.878-0.976	0.004

HR, hazard ratio; CI, confidence interval; TBIL, total bilirubin; HDL, high-density lipoprotein cholesterol; LDL, low-density lipoprotein cholesterol. Model 1: univariable analysis. Model 2: adjusted for age and gender. Model 3: adjusted for age, gender, eGFR, UA, proteinuria, diabetes, hypertension. Model 4: adjusted for age, gender, eGFR, UA, proteinuria, diabetes, hypertension and Oxford MEST-C pathological scores.

To clarify the biological interpretation of the bilirubin-lipid ratios, we separately evaluated TBIL and each lipid parameter in multivariable Cox models. As shown in **Supplementary Table S2**, TBIL alone was significantly associated with a reduced risk of the composite renal outcome in the fully adjusted model (HR = 0.960, 95% CI: 0.935–0.986, P = 0.003). Among individual lipid parameters, only LDL showed a modest positive association with the outcome (HR = 1.056, 95% CI: 1.001–1.114, P = 0.044), whereas triglycerides, cholesterol, and HDL were not significantly associated (all P > 0.05). We further evaluated the incremental predictive value of the ratios using C-index, NRI, and IDI (**Supplementary Table S3**). The addition of TBIL alone improved the C-index from 0.699 to 0.7065, while the corresponding ratios showed slightly lower C-indices (0.7023–0.7057). The ΔC-index, NRI, and IDI for the ratios compared with the base model or individual components did not reach statistical significance (all P > 0.05).

### 3.3 Proportional hazards assumption and time-dependent effect of bilirubin-lipid ratios

We formally tested PH assumption using Schoenfeld residual tests. As shown in **Table 3**, the PH assumption was violated for all four bilirubin-lipid ratios (all P < 0.05), indicating that the protective effects of these ratios varied over time. To address this violation and to assess the stability of our primary findings, we performed two complementary sensitivity analyses. In time-dependent Cox models, all four ratios remained significantly protective (all P < 0.001): TBIL/Triglyceride (HR = 0.910, 95% CI: 0.863–0.960), TBIL/Cholesterol (HR = 0.630, 95% CI: 0.501–0.792), TBIL/HDL (HR = 0.903, 95% CI: 0.853–0.956), and TBIL/LDL (HR = 0.816, 95% CI: 0.729–0.914). Moreover, we conducted piecewise Cox models stratified at the median follow-up time of 3.13 years. In the early phase (≤3.13 years), all ratios demonstrated significant protective associations: TBIL/Triglyceride (HR = 0.944, 95% CI: 0.900–0.991, P = 0.019), TBIL/Cholesterol (HR = 0.694, 95% CI: 0.559–0.863, P = 0.001), TBIL/HDL (HR = 0.911, 95% CI: 0.860–0.965, P = 0.001), and TBIL/LDL (HR = 0.844, 95% CI: 0.756–0.941, P = 0.002). In the late phase (>3.13 years), the protective effects were attenuated and no longer statistically significant for any of the four ratios (all P > 0.05).

**Table 3:** Proportional hazards assumption testing and time-dependent effect of bilirubin-lipid ratios.

	PH test P*	Time-dependent Cox model†		Piecewise Cox model (3.13-year cutpoint)‡			
		HR (95% CI)	P	≤3.13 years HR (95% CI)	P	>3.13 years HR (95% CI)	P
TBIL×Triglyceride	0.004	0.910 (0.863-0.960)	<0.001	0.944 (0.900-0.991)	0.019	0.983 (0.955-1.011)	0.229
TBIL×Cholesterol	0.004	0.630 (0.501-0.792)	<0.001	0.694 (0.559-0.863)	0.001	0.914 (0.812-1.028)	0.134
TBIL×HDL	0.004	0.903 (0.853-0.956)	<0.001	0.911 (0.860-0.965)	0.001	0.992 (0.964-1.020)	0.562
TBIL×LDL	0.015	0.816 (0.729-0.914)	<0.001	0.844 (0.756-0.941)	0.002	0.957 (0.903-1.015)	0.143

*Schoenfeld residual test for the proportional hazard assumption for each ratio. †Time-dependent Cox model with Time × Ratio interaction term. ‡Piecewise Cox models stratified at the median follow-up time of 3.13 years. All models were adjusted for age, gender, eGFR, UA, proteinuria, diabetes, hypertension, and Oxford MEST-C pathological scores.

### 3.4 Dose–response associations and optimal cut-off values of bilirubin-lipid ratios for the composite renal outcome

RCS analyses were performed to assess potential nonlinear associations between bilirubin-lipid ratios and the risk of composite renal outcome (**Figure 1**). Based on the AIC values (**Supplementary Table S5**), the 3-knot model was selected for TBIL/Triglyceride, TBIL/Cholesterol, and TBIL/HDL, while the 4-knot model was selected for TBIL/LDL. Knots were placed at the 10th, 50th, and 90th percentiles for the 3-knot models, and at the 5th, 35th, 65th, and 95th percentiles for the 4-knot model, following Harrell’s recommendations. After full adjustment for age, gender, eGFR, serum uric acid, proteinuria, diabetes, hypertension, and Oxford MEST-C scores, all four ratios exhibited a consistent downward trend in HR as their levels increased, indicating a progressively lower risk of the composite renal outcome with higher ratio values. TBIL/Triglyceride, TBIL/Cholesterol, and TBIL/HDL demonstrated predominantly linear inverse associations (P for nonlinearity = 0.5792, 0.5453, 0.9560, respectively). In contrast, TBIL/LDL showed evidence of a nonlinear relationship (P for nonlinearity = 0.0499), characterized by a threshold-saturation pattern. The hazard ratio for TBIL/LDL crossed unity (HR = 1) at approximately 2.12. Below this threshold, higher TBIL/LDL levels were associated with increased risk (HR > 1), whereas above this threshold, progressively higher levels were associated with reduced risk, with the protective effect plateauing at higher values. To explore optimal cut-off values for clinical risk stratification, we performed ROC curve analyses based on the Youden index. The optimal cut-off values for TBIL/Triglyceride, TBIL/Cholesterol, TBIL/HDL, and TBIL/LDL were 10.380, 1.206, 7.943, and 2.319, respectively (**Supplementary Table S4**).

**Figure 1:**
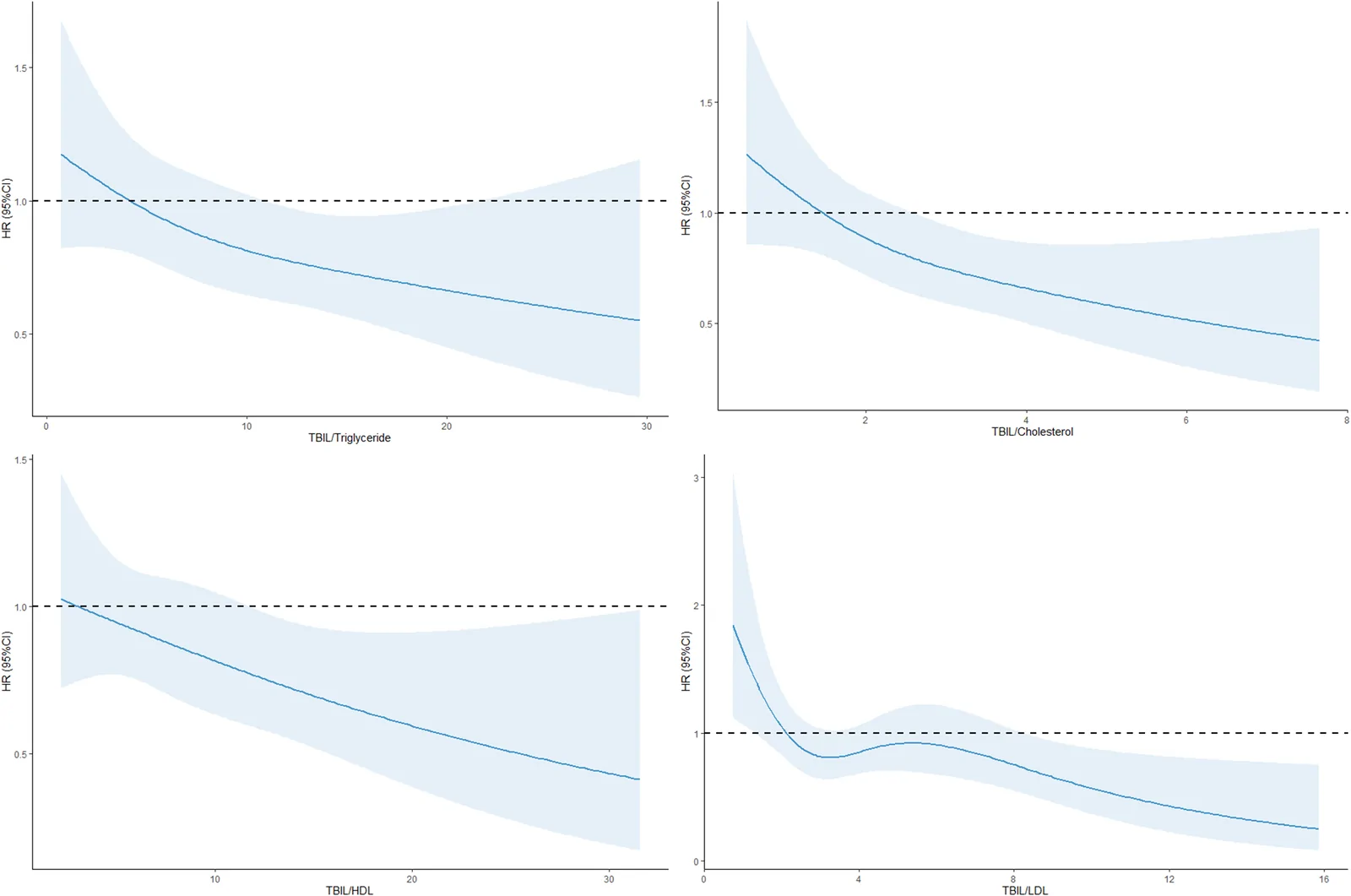
Restricted cubic spline curves showing the associations between bilirubin-lipid ratios and the risk of the composite renal outcome. Adjusted for age, gender, eGFR, UA, proteinuria, diabetes, hypertension and Oxford MEST-C pathological scores.

### 3.5 Subgroup analyses of the associations between bilirubin-lipid ratios and composite renal outcome

Subgroup analyses were conducted to evaluate the consistency of the prognostic associations across clinically relevant strata, including age, gender, comorbidities (hypertension, diabetes mellitus), lifestyle factors (smoking, alcohol consumption), treatment regimens (best supportive care, glucocorticoids, immunosuppressant/targeted drugs), and Oxford classification (MEST-C scores, **Figure 2**). All models were adjusted for the same covariates as in Model 4. Overall, the inverse associations between higher bilirubin-lipid ratios and the composite renal outcome were consistent across most subgroups.

**Figure 2:**
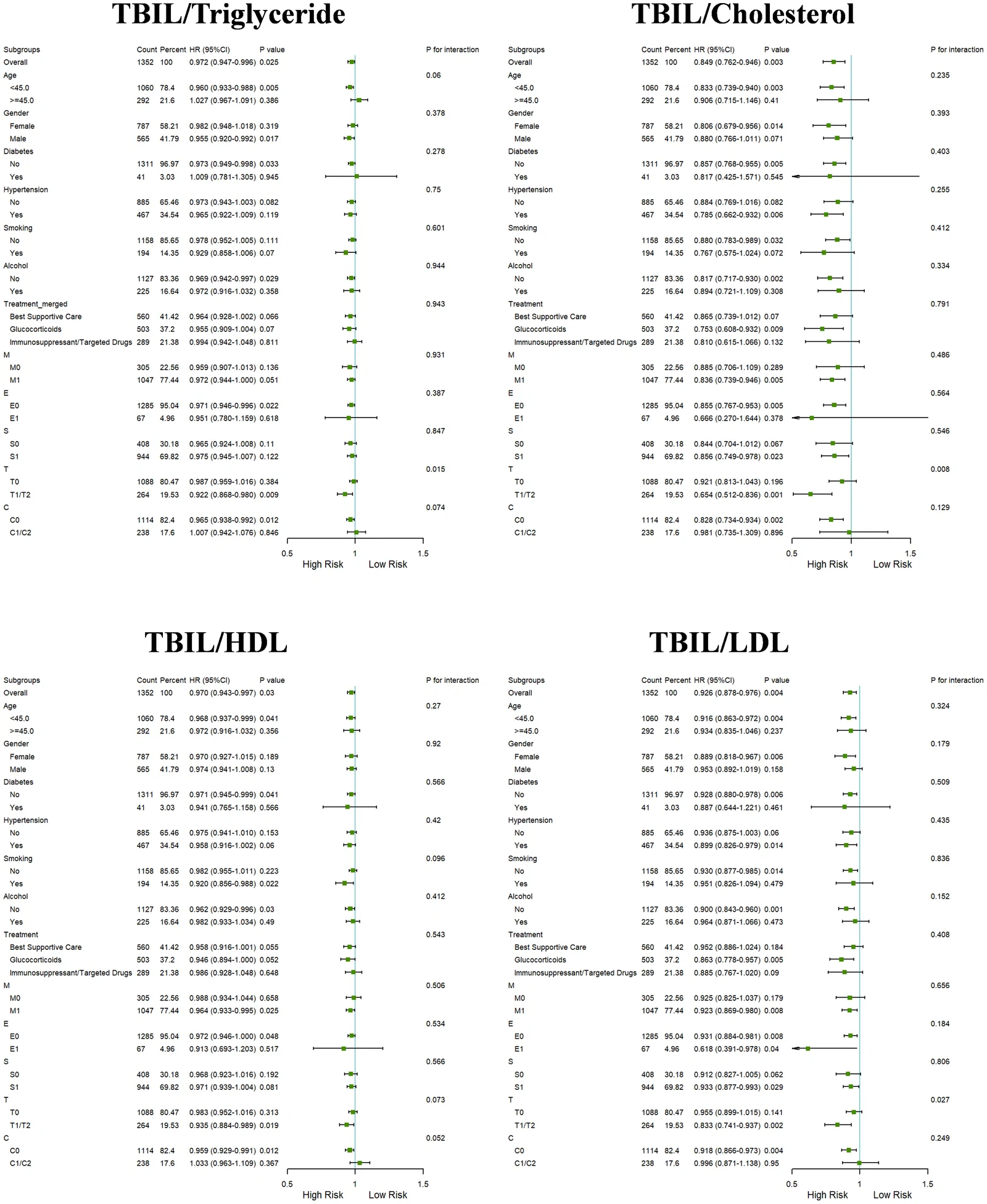
Subgroup analyses of the associations between bilirubin-lipid ratios and the composite renal outcome.

Notably, significant interactions were observed with the Oxford T score (tubular atrophy/interstitial fibrosis). The protective effects of TBIL/Triglyceride (P for interaction = 0.015), TBIL/Cholesterol (P for interaction = 0.008), and TBIL/LDL (P for interaction = 0.027) were significantly modified by the T score, with TBIL/HDL showing a similar but nonsignificant trend (P for interaction = 0.073). Specifically, the inverse associations were more pronounced in patients with advanced tubulointerstitial lesions (T1/T2): TBIL/Triglyceride (HR = 0.922, P = 0.009), TBIL/Cholesterol (HR = 0.654, P = 0.001), TBIL/HDL (HR = 0.935, P = 0.019), and TBIL/LDL (HR = 0.833, P = 0.002). In contrast, none of the four ratios reached statistical significance in the T0 subgroup (all P > 0.05).

Regarding age, although formal interaction tests were not statistically significant for any of the four ratios (all P for interaction > 0.05), the protective associations appeared stronger in younger patients. In the subgroup aged < 45 years, all ratios were significantly associated with reduced risk: TBIL/Triglyceride (HR = 0.960, P = 0.005), TBIL/Cholesterol (HR = 0.833, P = 0.003), TBIL/HDL (HR = 0.968, P = 0.041), and TBIL/LDL (HR = 0.916, P = 0.004). These associations were not statistically significant in patients aged ≥ 45 years (all P > 0.05). No significant effect modifications were detected for gender, hypertension, smoking, alcohol consumption, diabetes, or treatment regimen. The protective associations remained consistent across these strata, supporting the generalizability of the prognostic value of bilirubin-lipid ratios in IgAN.

### 3.6 Baseline bilirubin-lipid ratios and longitudinal eGFR trajectories

LME models were used to examine the longitudinal associations between baseline bilirubin-lipid ratios and repeated eGFR measurements over time (**Table 4**). As expected, a significant decline in eGFR was observed during follow-up across all models, with time coefficients ranging from –2.255 to –2.416 (all P for the “Time” < 0.001). After adjustment for clinical and pathological confounders, all four baseline bilirubin-lipid ratios were positively associated with longitudinal eGFR levels. Specifically, higher baseline values of TBIL/Triglyceride (Estimate = 0.318, 95% CI: 0.120 - 0.516, P = 0.002), TBIL/Cholesterol (Estimate = 1.242, 95% CI: 0.401 - 2.082, P = 0.004), TBIL/HDL (Estimate = 0.289, 95% CI: 0.079 to 0.499, P = 0.007), and TBIL/LDL (Estimate = 0.446, 95% CI: 0.041 - 0.851, P = 0.031) were associated with better preserved renal function over time. To assess whether these associations changed over time, interaction terms between time and each ratio (Time × Ratio) were evaluated. None of the interaction terms reached statistical significance, indicating that the protective associations of the bilirubin-lipid ratios with eGFR remained stable throughout the follow-up period, without significant attenuation or amplification.

**Table 4:** Linear mixed-effects models for the longitudinal association between bilirubin-lipid ratios and eGFR.

	Estimate	95%CI-lower	95%CI-upper	P-value
(Intercept)	158.261	151.287	165.234	<0.001
time	-2.324	-2.612	-2.035	<0.001
TBIL/Triglyceride	0.318	0.120	0.516	0.002
time:TBIL/Triglyceride	-0.001	-0.028	0.027	0.967
(Intercept)	159.408	152.619	166.198	<0.001
time	-2.362	-2.694	-2.029	<0.001
TBIL/Cholesterol	1.242	0.401	2.082	0.004
time:TBIL/Cholesterol	0.014	-0.105	0.133	0.821
(Intercept)	161.067	154.579	167.556	<0.001
time	-2.255	-2.563	-1.948	<0.001
TBIL/HDL	0.289	0.079	0.499	0.007
time:TBIL/HDL	-0.008	-0.039	0.023	0.600
(Intercept)	160.810	154.148	167.472	<0.001
time	-2.416	-2.724	-2.107	<0.001
TBIL/LDL	0.446	0.041	0.851	0.031
time:TBIL/LDL	0.020	-0.039	0.079	0.509

CI, confidence interval; TBIL, total bilirubin; HDL, high-density lipoprotein cholesterol; LDL, low-density lipoprotein cholesterol. Adjusted for age, gender, eGFR, UA, proteinuria, diabetes, hypertension and Oxford MEST-C pathological scores..

## 4 Discussion

This retrospective cohort study systematically evaluated the prognostic value of four serum bilirubin-lipid ratios (TBIL/Triglyceride, TBIL/Cholesterol, TBIL/HDL, and TBIL/LDL) in patients with biopsy-proven IgAN. The primary focus was on their associations with the composite renal outcome, defined as a ≥30% decline in eGFR or progression to ESRD, as well as longitudinal eGFR trajectories. Several key findings emerged from this analysis. First, higher baseline levels of all four bilirubin-lipid ratios were independently associated with a reduced risk of the composite renal outcome, even after adjustment for clinical and Oxford MEST-C pathological risk factors. Although the PH assumption was formally violated for all four ratios (Schoenfeld test P < 0.05), time-dependent Cox and piecewise Cox models confirmed that the protective effects remained significant during early follow-up (≤3.13 years) before attenuating thereafter. Second, RCS analyses demonstrated inverse dose-response relationships for all ratios, with TBIL/LDL exhibiting a nonlinear threshold-saturation pattern. Third, subgroup analyses revealed that the inverse associations were more pronounced in patients with advanced tubulointerstitial lesions (Oxford T1/T2). Finally, LME models indicated that higher baseline ratios correlated with better preserved overall eGFR levels throughout the follow-up, but not with a significantly different slope of eGFR decline. Collectively, these findings suggest that serum bilirubin-lipid ratios may be useful prognostic biomarkers in IgAN, potentially reflecting the balance between endogenous antioxidant capacity and lipid-related renal injury.

The inverse associations between bilirubin-lipid ratios and adverse renal outcomes observed in this study are consistent with accumulating evidence supporting the renoprotective roles of bilirubin and the deleterious effects of dyslipidemia. The pathogenesis of IgAN involves a multi-hit process in which mesangial deposition of galactose-deficient IgA1 immune complexes triggers localized inflammation and oxidative stress (1, 2).Bilirubin, a potent endogenous antioxidant, scavenges reactive oxygen species, inhibits lipid peroxidation, and attenuates inflammatory responses implicated in kidney injury (21, 22). Recent studies in IgAN patients have reported that mildly elevated bilirubin levels are associated with milder clinical manifestations, less severe pathological damage, and improved renal outcomes (23–25). Conversely, dyslipidemia has been established as an independent risk factor for renal function deterioration through mechanisms involving direct cellular injury, glomerulosclerosis, and interstitial fibrosis (26). A recent study demonstrated that TBIL was inversely correlated with total cholesterol, triglycerides, and LDL in IgAN patients (27). Additionally, experimental evidence from a streptozotocin-induced diabetic nephropathy model showed that bilirubin treatment reduced serum lipid levels (28). By integrating antioxidant capacity (TBIL) and lipid burden, these composite ratios may better reflect the underlying pathophysiological balance than either component alone.

TBIL/Cholesterol consistently demonstrated a strong protective association with both the composite renal outcome and eGFR preservation. A recent retrospective cohort study similarly showed that a higher TBIL/Cholesterol ratio was associated with reduced CKD progression in patients with type 2 diabetes (6). Mechanistically, cholesterol accumulation has been associated with renal injury through multiple interconnected pathways, including oxidative stress, inflammation, apoptosis, and fibrogenic remodeling (29). Experimental studies further suggest that hypercholesterolemia may promote intrarenal profibrotic signaling and extracellular matrix deposition, particularly in the tubulointerstitial compartment, which has been associated with renal functional decline (30). Additionally, hypercholesterolemia may be related to glomerular endothelial injury through increased cellular cholesterol deposition due to ATP-binding cassette transporter A1 (ABCA1) deficiency, thereby contributing to inflammation and apoptosis (31, 32).

TBIL/Triglyceride, TBIL/HDL, and TBIL/LDL also demonstrated renoprotective effects, although their prognostic performance was weaker than that of TBIL/Cholesterol. Experimental studies have shown that bilirubin inhibits key regulators and enzymes involved in triglyceride synthesis, including liver X receptor alpha (LXRα), sterol regulatory element-binding protein 1 (SREBP-1), stearoyl-CoA desaturase 1 (SCD-1) and fatty acid synthase (FAS) (28, 33, 34). This suggests that bilirubin’s regulatory effect on triglycerides involves direct inhibition of lipid synthesis pathways rather than solely indirect antioxidant effects. Hypertriglyceridemia has been identified as an independent risk factor for glomerulosclerosis, mesangial cell proliferation, mesangial matrix expansion, tubular atrophy, and interstitial fibrosis in IgAN (35, 36). Similarly, elevated LDL has been linked to ESRD in IgAN (37), potentially through downregulation of Klotho, a renoprotective protein (38). Oxidized and glycated LDL have been associated with mesangial cell proliferation by upregulating fibronectin and laminin, processes closely linked to IgAN pathogenesis (39).

The findings regarding TBIL/HDL present an apparent paradox. HDL is traditionally regarded as protective, yet a higher TBIL/HDL ratio was associated with lower renal risk in our analyses. This may be explained by qualitative changes in HDL (“dysfunctional HDL”) rather than its quantity (40). In CKD, HDL undergoes structural and functional alterations (41, 42). These are associated with impaired HDL cholesterol efflux capacity, antioxidant properties, and endothelial protection (41, 43, 44). This may partly explain the apparent discrepancy whereby TBIL/HDL showed protective associations in Cox, RCS, and LME analyses but did not yield significant separation in Kaplan–Meier curves. Moreover, the Kaplan–Meier method is a non-parametric, univariable procedure that requires dichotomization of continuous variables; stratifying TBIL/HDL at its median discards quantitative information and reduces statistical power (45). Cox regression and RCS models handle TBIL/HDL as a continuous variable across its full range, while LME models capture longitudinal eGFR trajectories, all with adjustment for established confounders (46). These methodological advantages enable the detection of a graded, covariate-adjusted relationship that a median-based binary split cannot reveal.

Although the C-index of the ratios was slightly lower than that of TBIL alone and the ΔC-index, NRI, and IDI did not reach statistical significance, the ratios may still be clinically informative. These metrics assess incremental predictive performance within a specific model (47), but may not fully capture the value of composite biomarkers that reflect physiological balance. **Supplementary Table S2** showed that TBIL alone had a modest effect (HR = 0.960), individual lipid parameters were largely non-significant, whereas all four ratios demonstrated consistent protective effects, particularly TBIL/Cholesterol (**Table 2**, HR = 0.849). Indeed, the ratios synthesize the opposing contributions of antioxidant capacity and lipid burden into a single metric, which is especially pertinent to IgAN pathogenesis, where both oxidative stress and lipotoxicity actively drive disease progression (6, 23, 27, 48). This framework parallels established ratio-based biomarkers such as the neutrophil-to-lymphocyte ratio, reflecting physiological equilibrium rather than absolute values, and these readily available, cost-effective ratios warrant further validation as practical adjuncts for risk stratification in IgAN.

To explore potential cut-offs for clinical risk stratification, we calculated the optimal cut-off values for each ratio using the Youden index based on ROC curves (49). However, the AUC values ranged from 0.536 to 0.547, indicating that these ratios have limited discriminative ability when used as single binary classifiers. This is not unexpected, as the bilirubin-lipid ratios are continuous integrative biomarkers reflecting the dynamic balance between antioxidant capacity and lipid burden, and a single dichotomized threshold may not fully capture their prognostic information (50). Similarly, the TBIL/LDL threshold of approximately 2.12 identified in the RCS analysis should be interpreted as a model-derived statistical transition point rather than a clinically validated cutoff. The difference between the RCS-derived value (2.12) and the Youden index-derived value (2.32) further highlights the uncertainty of defining a single clinically meaningful threshold in the current dataset. Therefore, these cut-offs require validation in independent cohorts before clinical application.

A notable finding was the significant interaction between bilirubin-lipid ratios and the Oxford T score (P for interaction < 0.05 for TBIL/Triglyceride, TBIL/Cholesterol, and TBIL/LDL). The protective associations were most pronounced in patients with advanced tubulointerstitial lesions (T1/T2) but lost statistical significance in those without tubular atrophy (T0). Tubular atrophy and interstitial fibrosis represent the final common pathway of progressive kidney injury, driven largely by chronic inflammation and oxidative stress (2, 51). Lipid accumulation in proximal tubular cells has been increasingly recognized as a contributor to tubulointerstitial injury and lipotoxicity (48, 52). Our findings suggest that these ratios may be more informative in patients with more advanced tubulointerstitial damage, although this observation requires confirmation.

To our knowledge, this is the first study to systematically evaluate and compare the prognostic value of four complementary bilirubin-lipid ratios in a large, well-characterized cohort of biopsy-proven IgAN patients. These ratios are simple, inexpensive, and widely available, as they rely only on routine laboratory tests, making them attractive candidates for prognostic screening in clinical practice. By integrating the protective effects of bilirubin with the pathogenic effects of lipids, they may provide a more comprehensive reflection of renal risk than either component alone. Importantly, the CIE analysis showed that inclusion of candidate covariates produced only minimal changes in the HR (all <10%), suggesting that the observed protective associations were not substantially confounded by traditional risk factors. This robustness suggests that these composite ratios capture pathophysiological information largely independent of established clinical, laboratory, and pathological markers, supporting their potential utility as practical risk stratification tools in IgAN.

However, several limitations should be acknowledged. First, our analyses relied solely on baseline laboratory measurements. Since bilirubin and lipid profiles fluctuate over time, future studies incorporating time-varying covariates are warranted to capture their longitudinal impact more accurately. Second, we did not measure inflammatory or oxidative stress markers (e.g. C-reactive protein, malondialdehyde), which could provide additional mechanistic insights. Third, despite extensive multivariable adjustment, including baseline use of RAAS inhibitors, SGLT2 inhibitors, and lipid-lowering therapy, residual confounding from unmeasured factors such as statin intensity, dietary habits, or treatment changes over time cannot be entirely ruled out—particularly given the extended enrolment period spanning 2009 to 2025 and the lack of captured longitudinal treatment adjustments during follow-up. Fourth, we did not systematically collect data on specific hepatobiliary diseases or hemolytic disorders, which may influence serum bilirubin levels independently of renal antioxidant mechanisms. Fifth, the median follow-up duration of 3.13 years, although sufficient to detect early renal outcomes and comparable to that in landmark studies such as TESTING and the Toronto cohort (53, 54), remains relatively short for IgAN. Future studies with extended follow-up are warranted to validate the long-term prognostic value of these bilirubin-lipid ratios. Finally, as a single-center retrospective study conducted in a Chinese cohort, the generalizability of our findings is limited. External validation in independent and multicenter cohorts will be necessary before broader clinical application.

In conclusion, higher serum bilirubin-lipid ratios, particularly TBIL/Cholesterol, were independently associated with a lower risk of adverse renal outcomes and better preserved longitudinal renal function in patients with IgAN. These findings suggest that bilirubin-lipid ratios may provide incremental prognostic information beyond conventional clinical and pathological indicators and may help improve risk stratification in this population.

## Data Availability

The raw data supporting the conclusions of this article will be made available by the authors, without undue reservation.
