# Supplementary material for: Prognostic value of serum total bilirubin-lipid ratios in patients with IgA nephropathy
Source: Front Endocrinol (Lausanne). 2026 Sep 16;17:1898387. doi: 10.3389/fendo.2026.1898387 (PMC13623543; doi:10.3389/fendo.2026.1898387)
Supplement: Supplementary file 1 [file Supplementaryfile1.docx]

***Supplementary Material***

Supplementary Table S1. Sensitivity analyses of the association between bilirubin-lipid ratios and the composite renal outcome.

|  | HR | 95%CI | P value |
| --- | --- | --- | --- |
| Exclusion of patients with follow-up duration < 1 year* | | | |
| TBIL/Triglyceride | 0.976 | 0.951-1.001 | 0.058 |
| TBIL/Cholesterol | 0.863 | 0.774-0.963 | 0.008 |
| TBIL/HDL | 0.971 | 0.943-0.999 | 0.043 |
| TBIL/LDL | 0.933 | 0.884-0.984 | 0.011 |
| Exclusion of patients with baseline eGFR < 30 mL/min/1.73 m²* | | | |
| TBIL/Triglyceride | 0.978 | 0.954-1.003 | 0.083 |
| TBIL/Cholesterol | 0.875 | 0.786-0.975 | 0.015 |
| TBIL/HDL | 0.975 | 0.948-1.003 | 0.076 |
| TBIL/LDL | 0.938 | 0.889-0.989 | 0.017 |
| Exclusion of patients with baseline proteinuria < 0.5 g/24h* | | | |
| TBIL/Triglyceride | 0.96 | 0.934-0.988 | 0.005 |
| TBIL/Cholesterol | 0.821 | 0.730-0.924 | 0.001 |
| TBIL/HDL | 0.962 | 0.934-0.992 | 0.012 |
| TBIL/LDL | 0.913 | 0.862-0.968 | 0.002 |
| Exclusion of patients receiving baseline lipid-lowering therapy* | | | |
| TBIL/Triglyceride | 0.979 | 0.954-1.006 | 0.121 |
| TBIL/Cholesterol | 0.866 | 0.774-0.970 | 0.013 |
| TBIL/HDL | 0.971 | 0.943-1.000 | 0.052 |
| TBIL/LDL | 0.932 | 0.881-0.985 | 0.013 |
| Adjusted for age, gender, eGFR, UA, proteinuria, diabetes, hypertension, Oxford MEST-C pathological scores, lipid-lowering therapy, RAAS inhibitors and SGLT2 inhibitors. | | | |

| TBIL/Triglyceride | 0.971 | 0.947-0.996 | 0.021 |
| --- | --- | --- | --- |
| TBIL/Cholesterol | 0.838 | 0.751-0.936 | 0.002 |
| TBIL/HDL | 0.966 | 0.940-0.994 | 0.018 |
| TBIL/LDL | 0.921 | 0.873-0.972 | 0.003 |
| Adjusted for age, gender, eGFR, UA, proteinuria, diabetes, hypertension, Oxford MEST-C pathological scores, ALT and AST. | | | |
| TBIL/Triglyceride | 0.971 | 0.946-0.996 | 0.022 |
| TBIL/Cholesterol | 0.845 | 0.758-0.943 | 0.003 |
| TBIL/HDL | 0.969 | 0.942-0.997 | 0.029 |
| TBIL/LDL | 0.924 | 0.876-0.975 | 0.004 |

Abbreviations: HR, hazard ratio; CI, confidence interval; TBIL, total bilirubin; HDL, high-density lipoprotein cholesterol; LDL, low-density lipoprotein cholesterol; eGFR, estimated glomerular filtration rate; ALT, alanine aminotransferase; AST, aspartate aminotransferase; RAAS, renin‑angiotensin‑aldosterone system; SGLT2, sodium-glucose cotransporter 2. *Adjusted for age, gender, eGFR, UA, proteinuria, diabetes, hypertension and Oxford MEST-C pathological scores.

Supplementary Table S2. Association of TBIL and individual lipid parameters with the composite renal outcome.

| Model | HR | 95%CI | P value |
| --- | --- | --- | --- |
| TBIL |  |  |  |
| Model1 | 0.955 | 0.930-0.980 | 0.001 |
| Model2 | 0.952 | 0.927-0.978 | 0.000 |
| Model3 | 0.961 | 0.936-0.987 | 0.003 |
| Model4 | 0.960 | 0.935-0.986 | 0.003 |
| Triglyceride | |  |  |
| Model1 | 1.057 | 0.978-1.143 | 0.163 |
| Model2 | 1.029 | 0.948-1.117 | 0.496 |
| Model3 | 0.988 | 0.902-1.082 | 0.790 |
| Model4 | 1.001 | 0.914-1.095 | 0.988 |
| Cholesterol | |  |  |
| Model1 | 1.060 | 0.984-1.142 | 0.125 |
| Model2 | 1.050 | 0.972-1.133 | 0.214 |
| Model3 | 1.023 | 0.936-1.118 | 0.616 |
| Model4 | 1.034 | 0.944-1.132 | 0.475 |
| HDL |  |  |  |
| Model1 | 0.829 | 0.634-1.085 | 0.172 |
| Model2 | 0.874 | 0.661-1.157 | 0.348 |
| Model3 | 0.912 | 0.680-1.224 | 0.541 |
| Model4 | 0.863 | 0.643-1.158 | 0.325 |
| LDL |  |  |  |
| Model1 | 1.075 | 1.024-1.129 | 0.004 |
| Model2 | 1.064 | 1.012-1.119 | 0.015 |
| Model3 | 1.053 | 0.997-1.113 | 0.066 |
| Model4 | 1.056 | 1.001-1.114 | 0.044 |

Abbreviations: HR, hazard ratio; CI, confidence interval; TBIL, total bilirubin; HDL, high-density lipoprotein cholesterol; LDL, low-density lipoprotein cholesterol. Model 1: univariable analysis. Model 2: adjusted for age and gender. Model 3: adjusted for age, gender, eGFR, UA, proteinuria, diabetes, hypertension. Model 4: adjusted for age, gender, eGFR, UA, proteinuria, diabetes, hypertension and Oxford MEST-C pathological scores.

Supplementary Table S3. C‑index of individual components and bilirubin‑lipid ratios, and their incremental predictive value assessed by ΔC‑index, NRI, and IDI.

| Panel A. C-index of individual components and ratios | | | | | | |
| --- | --- | --- | --- | --- | --- | --- |
| Ratio | Base model | + TBIL | + Lipid* | + Ratio |  |  |
| TBIL/Triglyceride | 0.699 | 0.7065 | 0.699 | 0.7023 |  |  |
| TBIL/Cholesterol | 0.699 | 0.7065 | 0.6991 | 0.7057 |  |  |
| TBIL/HDL | 0.699 | 0.7065 | 0.6991 | 0.7035 |  |  |
| TBIL/LDL | 0.699 | 0.7065 | 0.6998 | 0.7056 |  |  |
| Panel B. Incremental predictive value of bilirubin-lipid ratios. | | | | | | |
|  | ΔC-index (95% CI) | P value | NRI (95% CI) | P value | IDI (95% CI) | P value |
| TBIL/Triglyceride vs Base | 0.0033 (-0.0013 - 0.0143) | 0.212 | 0.0630 (-0.1048 - 0.1727) | 0.780 | 0.0077 (-0.0116 - 0.0150) | 0.748 |
| TBIL/Triglyceride vs TBIL | -0.0041 (-0.0130 - 0.0049) | 0.380 | -0.0543 (-0.1767 - 0.1191) | 0.724 | -0.0051 (-0.0161 - 0.0093) | 0.512 |
| TBIL/Triglyceride vs Triglyceride | 0.0034 (-0.0032 - 0.0131) | 0.340 | 0.0630 (-0.1076 - 0.1845) | 0.884 | 0.0077 (-0.0131 - 0.0143) | 0.840 |
| TBIL/Cholesterol vs Base | 0.0067 (-0.0002 - 0.0190) | 0.064 | 0.0580 (-0.0774 - 0.1642) | 0.464 | 0.0137 (-0.0108 - 0.0225) | 0.384 |
| TBIL/Cholesterol vs TBIL | -0.0007 (-0.0066 - 0.0059) | 0.992 | 0.0313 (-0.0944 - 0.1663) | 0.812 | 0.0009 (-0.0078 - 0.0101) | 0.644 |
| TBIL/Cholesterol vs Cholesterol | 0.0066 (-0.0009 - 0.0169) | 0.092 | 0.0468 (-0.1286 - 0.1577) | 0.652 | 0.0128 (-0.0120 - 0.0194) | 0.532 |
| TBIL/HDL vs Base | 0.0045 (-0.0012 - 0.0154) | 0.124 | 0.0747 (-0.0510 - 0.2165) | 0.236 | 0.0100 (-0.0068 - 0.0211) | 0.264 |
| TBIL/HDL vs TBIL | -0.0030 (-0.0104 - 0.0027) | 0.396 | 0.0733 (-0.0820 - 0.1910) | 0.588 | -0.0028 (-0.0077 - 0.0125) | 0.692 |
| TBIL/HDL vs HDL | 0.0044 (-0.0061 - 0.0163) | 0.392 | 0.1175 (-0.0492 - 0.2132) | 0.316 | 0.0107 (-0.0079 - 0.0249) | 0.288 |
| TBIL/LDL vs Base | 0.0066 (0.0000 - 0.0195) | 0.052 | 0.0520 (-0.1191 - 0.1690) | 0.508 | 0.0106 (-0.0116 - 0.0214) | 0.368 |
| TBIL/LDL vs TBIL | -0.0008 (-0.0097 - 0.0077) | 0.976 | -0.0408 (-0.1263 - 0.1331) | 0.876 | -0.0023 (-0.0131 - 0.0124) | 0.716 |
| TBIL/LDL vs Lipid | 0.0058 (-0.0007 - 0.0164) | 0.104 | 0.0252 (-0.1187 - 0.1489) | 0.692 | 0.0095 (-0.0144 - 0.0170) | 0.600 |

Abbreviations: NRI, net reclassification improvement; IDI, integrated discrimination improvement; CI, confidence interval. The base model was adjusted for age, gender, eGFR, uric acid, proteinuria, diabetes, hypertension, and Oxford MEST-C pathological scores. All extended models were adjusted for the same covariates plus the indicated variable(s). *Lipid refers to the corresponding individual lipid parameter (triglyceride, cholesterol, HDL, or LDL, respectively). NRI and IDI were calculated at the median follow-up time of 3.13 years.

Supplementary Table S4. Optimal cut‑off values of bilirubin-lipid ratios for risk stratification based on the Youden index.

|  | Cutoff | AUC | Sensitivity | Specificity | Youden |
| --- | --- | --- | --- | --- | --- |
| TBIL/Triglyceride | 10.380 | 0.547 | 0.816 | 0.273 | 0.089 |
| TBIL/Cholesterol | 1.206 | 0.545 | 0.230 | 0.852 | 0.082 |
| TBIL/HDL | 7.943 | 0.536 | 0.622 | 0.446 | 0.068 |
| TBIL/LDL | 2.319 | 0.541 | 0.311 | 0.774 | 0.085 |

Abbreviations: TBIL, total bilirubin; HDL, high-density lipoprotein cholesterol; LDL, low-density lipoprotein cholesterol.

Supplementary Table S5. Knot selection for restricted cubic spline models based on Akaike information criterion.

|  | 3 knots | 4 knots | 5 knots | Selected knots |
| --- | --- | --- | --- | --- |
| TBIL/Triglyceride | 3349.925 | 3351.919 | 3353.392 | 3 |
| TBIL/Cholesterol | 3345.205 | 3345.164 | 3347.107 | 3 |
| TBIL/HDL | 3350.161 | 3351.911 | 3351.997 | 3 |
| TBIL/LDL | 3345.815 | 3342.155 | 3342.084 | 4 |

Abbreviations: TBIL, total bilirubin; HDL, high-density lipoprotein cholesterol; LDL, low-density lipoprotein cholesterol.

Supplementary Figure S1. Flow diagram of the study population.

Supplementary Figure S2. Kaplan–Meier curves for the composite renal outcome according to bilirubin-lipid ratio groups.

Abbreviations: TBIL, total bilirubin; HDL, high-density lipoprotein cholesterol; LDL, low-density lipoprotein cholesterol.

Supplementary Figure S3. Change-in-estimate (CIE) analysis of potential confounders in the associations between bilirubin-lipid ratios and the composite renal outcome in patients with IgAN.
